# Progression of Community-Acquired Pneumonia in a Morbidly Obese Patient With a History of Vaping and Asthma: A Multidisciplinary Approach to Management

**DOI:** 10.7759/cureus.57828

**Published:** 2024-04-08

**Authors:** Jennifer C Chiji-Aguma, Olaoluwa M Adeyemi, Akolade S Osanoto, Abiodun M Akanmode

**Affiliations:** 1 Family Medicine, Carle Bromenn Medical Center, Bloomington, USA; 2 Internal Medicine, Richmond Gabriel University, Kingstown, VCT; 3 Internal Medicine, Windsor University School of Medicine, St. Kitts, KNA; 4 Internal Medicine, Columbia University Medical Center, New York, USA

**Keywords:** lung abscess, empyema, asthma, morbid obesity, community-acquired pneumonia

## Abstract

Lung abscess and empyema represent significant complications of community-acquired pneumonia, particularly in patients with comorbidities such as obesity, asthma, and vaping (which can lead to vaping-associated lung injury). While these conditions rarely occur simultaneously, their coexistence significantly escalates both mortality and morbidity. Management strategies typically involve a multidisciplinary approach, incorporating diagnostic evaluation through imaging, administration of antibiotics, and often surgical drainage. While antibiotics are fundamental in treating both conditions, empyema management almost invariably necessitates surgical intervention. Initial imaging usually involves plain radiographs, although ultrasound and lung CT scans provide heightened sensitivity and fluid characterization. Here, we present the case of a 24-year-old morbidly obese patient with a history of bronchial asthma initially presenting with community-acquired pneumonia, which subsequently deteriorated into lung abscess and empyema, ultimately requiring surgical intervention.

## Introduction

Community-acquired pneumonia (CAP) poses significant challenges in management, especially in patients with underlying comorbidities such as obesity and asthma. These factors not only increase the risk of CAP but also contribute to severe complications and prolonged hospitalizations. Here, we present a case report documenting the clinical journey of a morbidly obese patient with a history of asthma and vaping, who experienced severe complications secondary to CAP.

Empyema, characterized by the accumulation of pus within the pleural space due to infiltrative pyogenic infection, is a known complication of CAP [1]. The mechanism underlying empyema development involves the transmigration of bacteria into the pleural space, often originating from adjacent lung infections or pneumonia [2]. Additionally, approximately 20%-57% of patients with pneumonia develop parapneumonic effusion, with a subset progressing to empyema [3].

A differential diagnosis that frequently presents a diagnostic challenge is lung abscess, wherein localized purulent infections become necrotic, resulting in cavities within the lung parenchyma [4]. Patients with both empyema and lung abscess demonstrate higher rates of ICU admission and overall mortality compared to those with empyema alone [5].

The standard treatment approach for empyema involves percutaneous or surgical drainage in conjunction with antibiotics, whereas lung abscesses are primarily managed with antibiotics alone, reserving drainage for complicated cases [6].

In this report, we present a case of a patient with concurrent right empyema and right lower lobe abscess. Our primary objective is to highlight the significance of recognizing the coexistence of both a lung abscess and empyema, emphasizing the critical need for prompt surgical intervention to optimize patient outcomes. 

Through this case report, we aim to enhance understanding of the clinical presentation, challenges, and management strategies associated with CAP in patients with a history of morbid obesity, asthma, and vaping history.

## Case presentation

Our case involves a 24-year-old man with a significant medical history, including asthma, vaping (nicotine), and morbid obesity (304 lbs), with a body mass index (BMI) of 42.2. Initially, he presented to a convenient care facility and was seen for a three-week history of upper respiratory tract symptoms, including congestion, sore throat, vomiting, and cough productive of sputum. His congestion improved over time with symptomatic treatment using over-the-counter (OTC) medication. He denied experiencing pleuritic chest pain, fevers, chills, or having any known exposure to sick contacts. Physical examination revealed normal respiratory effort and breath sounds. There was no evidence of respiratory distress, wheezing, stridor, rales, or chest tenderness, and the patient was eventually diagnosed with post-viral cough and prescribed benzonatate (Tessalon Perles).

About a week later, the patient returned to the convenient care facility reporting persistent productive cough with hemoptysis and a foul taste. He had no shortness of breath, pressure, postnasal drip, body aches, fever, or chills. His physical examination was unremarkable, with normal pulmonary effort and breath sounds.

A same-day outpatient chest x-ray was ordered, and treatment for pneumonia was initiated with Augmentin, Z-Pak, albuterol inhaler, prednisone, and OTC Mucinex. The patient was sent home with instructions to follow up with a primary care provider (PCP). 

**Figure 1:**
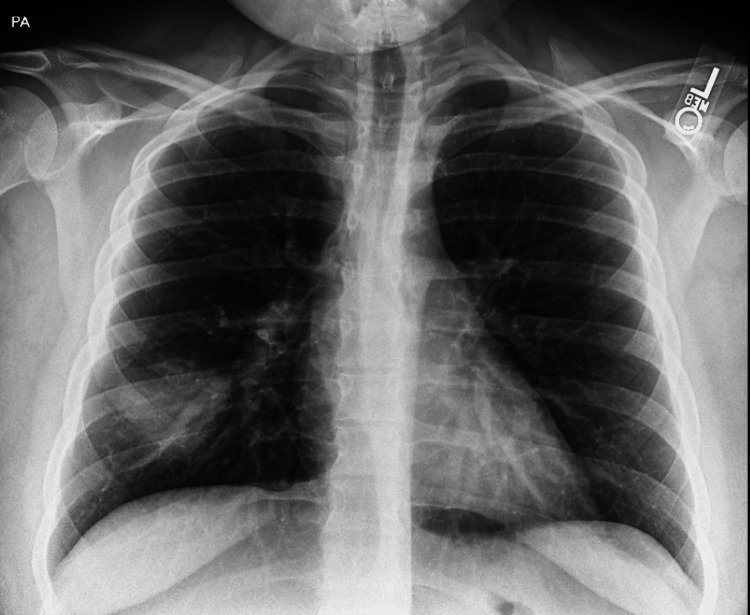
Right lower lobe mass-like opacity

An X-ray revealed a mass-like opacity in the right lower lobe (Figure 1), prompting a recommendation for further evaluation with a CT scan.

About six weeks after the initial visit to the convenient care facility, the patient presented to the emergency room with new-onset right-sided chest pain persisting for a week, exacerbated in the recumbent position. He also complained of associated shortness of breath, which was progressively worsening. On physical examination, the patient was in acute respiratory distress, presenting with tachycardia, scattered wheezing, decreased breath sounds bilaterally, and absent breath sounds at the right lung base. The patient was placed on 2 L of oxygen. He denied recent travel, outdoor activities, or exotic pets, but reported continued vaping multiple times per day despite his respiratory symptoms.

Routine blood work was ordered, and the patient met the PERC criteria, prompting a computed tomography angiography (CTA) PE chest scan along with pain control management. Laboratory findings were significant for leukocytosis, while other inflammatory markers were within normal limits. Empirical IV antibiotic therapy with Rocephin and azithromycin was initiated. 

**Figure 2:**
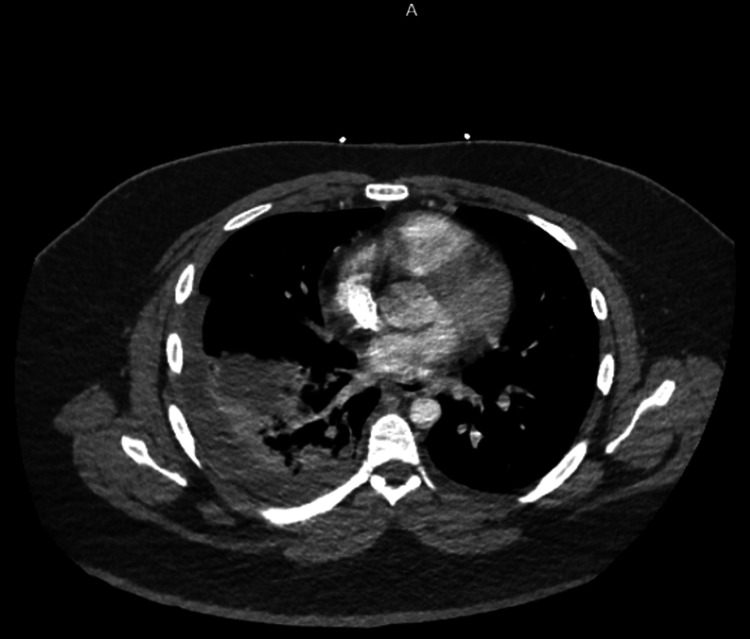
Chest CTA showing lobulated moderate left pleural effusion with consolidation in the right lower lobe, along with a mass-like consolidation in the superior segment of the right lower lobe CTA: Computed tomography angiography

The chest CTA revealed a lobulated moderate left pleural effusion with atelectasis and consolidation in the right lower lobe, along with a mass-like consolidation in the superior segment of the right lower lobe measuring approximately 3.6 by 3.4 cm proximally (Figure 2).

The patient was admitted for sepsis secondary to community-acquired pneumonia with a right-sided pleural effusion, concerning empyema vs. parapneumonic effusion, and the need to rule out a bronchogenic mass. Interventional radiology and Infectious Disease specialists were consulted, and labs were ordered for pleural fluid analysis and sputum culture, including a Gram stain.

Infectious Disease was consulted, and the recommendation was to continue empiric broad-spectrum antibiotics with vancomycin and Zosyn, with plans to follow up on cultures and urinary histoplasma antigen.

**Figure 3:**
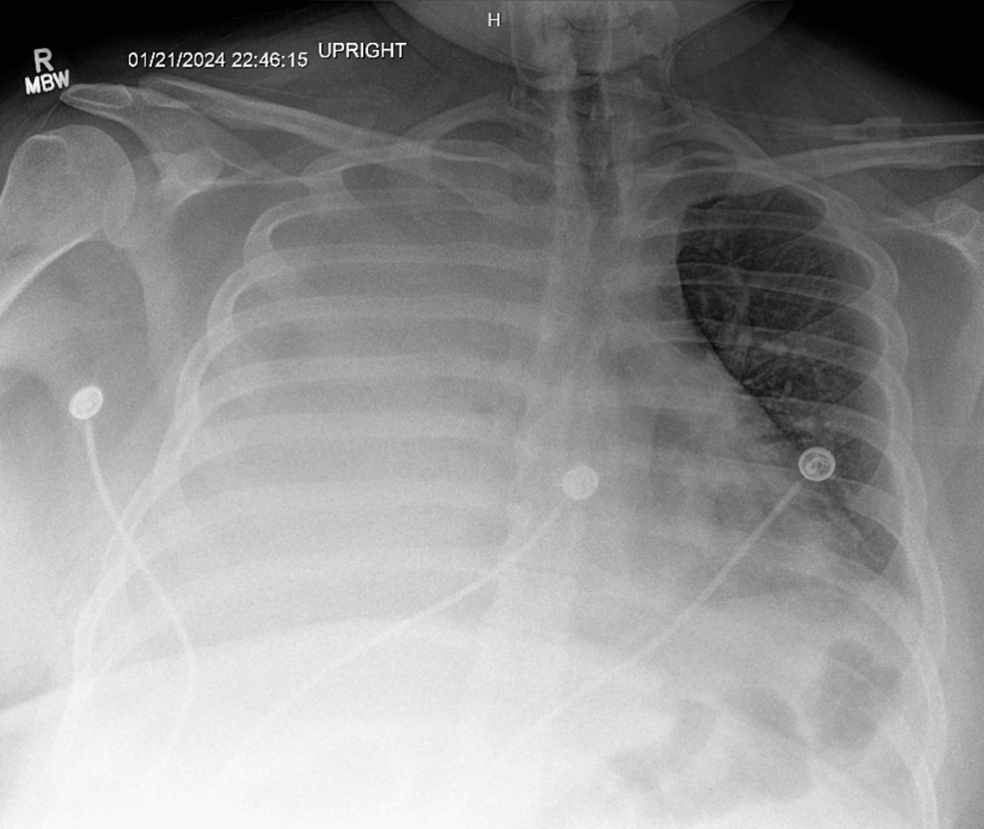
Near complete opacification of the right thorax

The patient experienced worsening shortness of breath, leading to an increase in oxygen to maintain saturations greater than 90%. A repeat chest X-ray showed complete opacification of the right lung (Figure 3). Arterial blood gas analysis revealed a pH of 7.33, pCO2 of 40.5, and a PO2 of 69.0 on a 10 L nasal cannula.

An ICU pulmonologist was consulted, and the patient was transferred to the ICU for close observation and further treatment. Bedside ultrasound revealed a free-flowing pleural effusion with septations. A recommendation was made for an interventional radiology-guided chest tube trial with tissue plasminogen activator (tPA) and dornase for three days, with plans to escalate to thoracic surgery for decortication if no improvement was observed.

The patient's oxygenation needs increased to vapotherm at 35 L with 75% FiO2. Azithromycin was changed to vancomycin. MRSA testing came back negative, and the respiratory panel was negative for flu, COVID-19, and respiratory syncytial virus (RSV), with negative Streptococcus and Legionella. Pleural fluid analysis, sputum culture, and Gram stain were also negative.

Due to a lack of IR on site for tPA administration and the worsening of the patient's condition, cardiothoracic surgery was consulted, and recommendations were for immediate right thoracotomy and decortication given multi-lobulated pleural processes and possible lung abscess.

Intraoperatively, the patient had an approximate 2 L of cloudy pleural effusion in the lung requiring complete decortication. The central aspect of the right lower lobe had a small abscess, which was marsupialized and drained. A 32-French chest tube was placed in the apex. A 28-French chest tube was placed at the level of the diaphragm. The patient tolerated the procedure well and was taken to the cardiovascular intensive care unit (CVICU) intubated and critically ill with a postoperative diagnosis of lung abscess, empyema, and morbid obesity.

**Figure 4:**
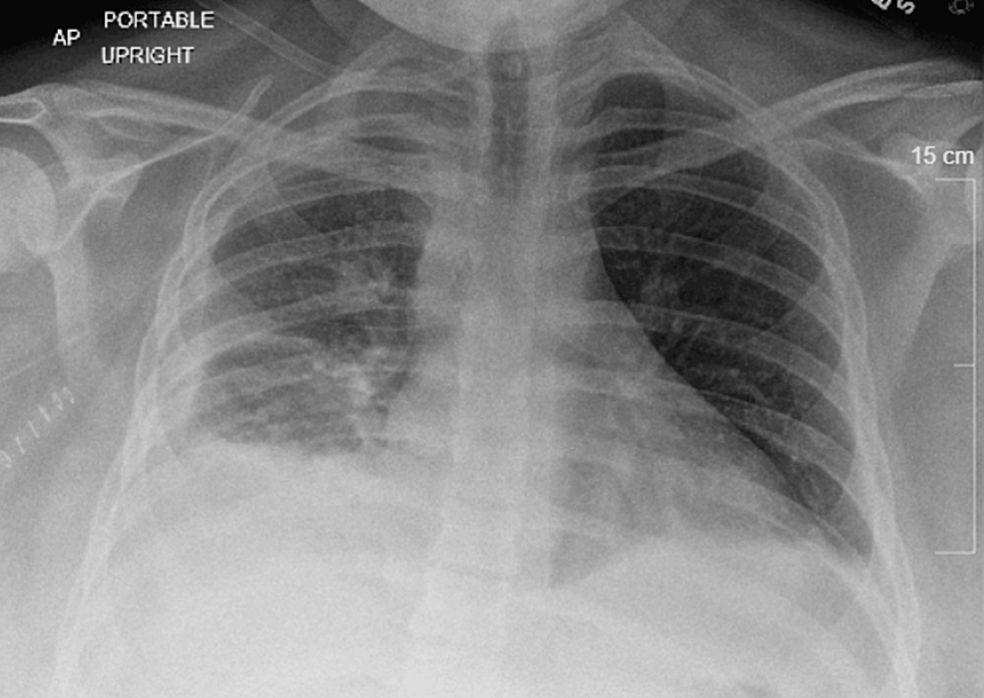
Partially improved aeration of the right hemithorax post thoracotomy

Following the postoperative period, a repeat chest X-ray revealed improved aeration, indicating positive progress (Figure 4). Subsequently, the patient underwent successful extubation within 24 hours. On physical exam, the patient was noted to be taking shallow breaths with no wheezing or rales, and improved aeration was noted throughout the lungs. Empiric broad-spectrum antibiotics with intravenous vancomycin and Zosyn was continued to maintain therapeutic management. His labs progressively improved, and cultures for urine, sputum, and blood, including Legionella antigen, were negative. The patient's oxygen requirement decreased to 2 L of oxygen, and he was eventually downgraded to the general floor. Subsequent cultures from drained fluid were negative for Streptococcus pneumoniae. 

Upon removal of the chest tube after six days, the patient was discharged with a peripherally inserted central catheter (PICC) line to facilitate continued intravenous administration of Zosyn at a dosage of 3.375 g every eight hours for a duration of four weeks from the initial dose.

Furthermore, a social worker was engaged to coordinate home health services for the patient's ongoing care needs. Additionally, the patient received counseling on vaping cessation as part of their comprehensive discharge plan.

Outpatient appointments were arranged for pulmonary function tests (PFTs) to address the patient's history of asthma and for a thorough workup for obstructive sleep apnea (OSA).

The patient's follow-up care was meticulously scheduled, with a visit to the PCP arranged within two to three days post discharge. Additionally, a subsequent follow-up appointment was scheduled in the cardiovascular clinic to occur one month after discharge to ensure continuity of care and to monitor the patient's progress.

## Discussion

Despite the widespread use of antibiotics and the availability of pneumococcal vaccines, empyema and lung abscesses remain significant sources of mortality and morbidity worldwide. Approximately one million admissions occur yearly for pneumonia, with 30-40% of cases developing parapneumonic effusions, and 5-10% of those progressing to empyema [7].

Empyema and lung abscesses represent two forms of pulmonary infections with overlapping symptoms; however, empyema tends to be more severe and has a complicated clinical course compared to lung abscesses. Differentiating between the two is crucial for appropriate clinical management, although simultaneous occurrence of both conditions is rare [6].

In spite of advances in chest imaging, a simple chest X-ray remains the initial modality, providing significant information. However, chest ultrasound, particularly point-of-care ultrasound (POCUS), and lung CT scans offer greater sensitivity for fluid detection and information about the pleural effusion's nature. Diagnostic thoracentesis is recommended for pleural effusions greater than 2 cm on a lateral decubitus film, with frank pus usually necessitating surgical drainage [8].

Ninety percent of all lung abscesses can be managed with antibiotic treatment alone, while 10% require interventional catheter or chest tubes, and only 1% require thoracic surgery due to complications independent of conservative or interventional treatment strategies [9]. Primary abscesses have an estimated mortality rate of less than 10%, while secondary abscesses, despite targeted antimicrobial therapy, are associated with a poor outcome, with factors such as old age, severe comorbidities, immunosuppression, bronchial obstruction, and neoplasms contributing significantly.

According to the American College of Chest Physicians (ACCP), empyema management aims to completely eradicate the infection through a combination of antimicrobials and pleural drainage via tube thoracostomy, with or without adjuvant intrapleural medications, video-assisted thoracoscopic surgery (VATS), or open thoracostomy with decortication. Surgical intervention due to conservative treatment failure is required in only 10% of patients, with a success rate of up to 90% and postoperative mortality rates of up to 33% [10-12].

In our patient, we suspected initial viral pneumonia, followed by secondary bacterial pneumonia weeks later. Hence, the opacity seen on the initial chest X-ray, likely a lung abscess, progressed into empyema noted on CTA scan imaging. Delayed follow-up despite persistent symptoms and comorbidities, including morbid obesity and bronchial asthma, likely contributed to rapid symptom progression. We also suspect vaping history contributed as e-cigarette or vaping-associated lung injury (EVALI) presents pulmonary manifestations, although the exact mechanism remains unclear [11].

## Conclusions

Managing empyema and lung abscesses requires timely diagnosis and an interdisciplinary management approach for a positive outcome. Patients with these conditions have higher ICU admission rates and overall higher 30-day mortality. Our case outlines medical and surgical management strategies for empyema and lung abscesses. Timely diagnosis and appropriate management of suspicious pulmonary lesions, especially in morbidly obese patients with other pulmonary complications such as bronchial asthma and vaping history, are crucial for mitigating poorer outcomes.
